# Allergic rhinitis children with obesity are more vulnerable to air pollution: a cross sectional study

**DOI:** 10.1038/s41598-023-30388-3

**Published:** 2023-03-04

**Authors:** Ruo-Ling Li, Chia-Ta Wu, Shan-Ming Chen, Ko-Huang Lue, Shiuan-Shinn Lee, Chang-Yao Tsao, Min-Sho Ku

**Affiliations:** 1 Department of Public Health, Institute of Public Health, Chung Shan Medical University, Taichung, Taiwan; 2 Department of Medical Management, Chung Shan Medical University Hospital, Taichung, Taiwan; 3 Department of Emergency Medicine, Changhua Christian Hospital, Changhua, Taiwan; 4 School of Medicine, Chung Shan Medical University, Taichung, Taiwan; 5 Division of Allergy, Asthma and Rheumatology, Department of Pediatrics, Chung Shan Medical University Hospital, Taichung, Taiwan; 6 School of Public Health, Chung Shan Medical University, Taichung, Taiwan; 7 Department of Internal Medicine, Chung Shan Medical University Hospital, Taichung, Taiwan

**Keywords:** Immunology, Environmental sciences, Diseases

## Abstract

The association between air pollution, allergic rhinitis (AR), and obesity has not been studied. From 2007 to 2011, 52 obese and 152 non-obese children (7–17 years old) with AR were recruited. Pediatric-Rhinoconjunctivitis-Quality-of-Life Questionnaire (PRQLQ) and nasal peak expiratory flow (NPEF) were tested. Association between the scores and rates of the two tests and mean air pollutant concentrations within 7 days before the tests were compared. When exposed to higher concentrations of CO, PM_10_, and PM_2.5_, the rates of worse nasal discomfort were 39.4%, 44.4% and 39.3% in obese children; and 18.0%, 21.9% and 19.7% in non-obese children, respectively. Compare to non-obese children, the rates in obese children were higher for CO (odds ratio (OR) 3.54, 95% confidence interval (CI) 1.15 ~ 10.92); PM_10_ (OR 3.26, 95% CI 1.01 ~ 10.57) and PM2.5 (OR 3.30; 95% CI 1.03 ~ 10.54). In obese children, correlations between higher concentrations of CO, PM_10_, PM_2.5_ and higher nasal discomfort (higher PRQLQ); and correlations between higher concentrations of CO, PM_10_, PM_2.5_, NMHC (non-methane hydrocarbon) and higher nasal mucosa inflammation (lower NPEF) were noted. Obesity negatively affected AR severity when AR children experienced higher concentrations of CO, PM_10_, and PM_2.5_. Increased nasal inflammation induced by air pollutants might be the underlying mechanism.

## Introduction

Recently, more attention has focused on the impact of air pollution on individual health. Obesity^1^ and allergic rhinitis (AR) are major public health issues worldwide. Therefore, determining the association between air pollution, obesity, and AR is crucial. However, this association has not been previously studied.

Regarding the relationship between air pollution and AR, epidemiological^2^ and biological^3^ studies had find their association. Regarding the relationship between obesity and AR, epidemiological^4,5^ and biological^6^ studies also find their association. Air pollution can induce the recruitment of inflammatory cells and inflammatory cytokines^7^. AR and obesity are considered chronic inflammatory illnesses^8,9^. Studies also found that the aforementioned inflammatory cells and cytokines induced by air pollutants are also increased in people with obesity or AR^10^. It indicated that air pollution, AR, and obesity share the same immune and inflammatory effects on the human body.

Therefore, it is reasonable to speculate that air pollutants might have a stronger adverse effect on the severity of AR when they are obese. However, only the separate relationship between air pollution and AR^2^; or AR and obesity^4,5^; or air pollution and obesity^11^ were studied. The combined associations between air pollution and obesity and AR were not studied. Therefore, a retrospective cross-sectional, case–control study was conducted to find the association. We aimed to investigate the influence of air pollutants on the severity of rhinitis (quality of life) and degree of nasal obstruction and nasal mucosa inflammation among AR children when they are obese or non-obese.

## Methods

### Air pollutants

The Environmental Protection Administration established five air pollution monitoring stations in Taichung, Taiwan. Concentrations of air pollutants, ambient temperature and relative humidity were measured continuously around the clock and reported hourly. Data on the mean daily levels of air pollutants, ambient temperature and relative humidity were supported and the data between 2007 and 2011 were gathered for the study. The following seven air pollutants were assessed: nitrogen dioxide (SO_2_), carbon monoxide (CO), particles ≤ 10 μm in diameter (PM_10_), particles ≤ 2.5 μm in diameter (PM_2.5_), nitric oxide (NO), nitric dioxide (NO_2_), and non-methane hydrocarbon (NMHC).

### Study population

From chart review, children aged 7–17 years with AR residing in Taichung, Taiwan, were recruited from a teaching hospital in the same city, from January 1, 2007, to December 31, 2011. AR was diagnosed by two pediatric allergy specialists. Based on the AR and its Impact on Asthma guidelines^12^, patients with moderate to severe AR were included. All patients underwent regular follow-ups at the hospital for at least a year. Those with congenital anomaly, congenital heart disease, chronic lung or other chronic disease and uncontrolled asthma were excluded. Those required antibiotics treatment or ever visited emergency room or admission for respiratory disease within 7 days before or after the tests were excluded. The weight and height of the participants (without shoes and outer clothing) were measured and recorded in the chart, and the body mass index (BMI, kg/m^2^) was calculated. Based on the growth charts for Taiwanese children and adolescents^13^, these participants were classified into (1) obese AR children (BMI > 85th percentile) group and (2) non-obese AR children (BMI < 85th percentile) group.

### Measurement of AR parameters

In these AR children, the Pediatric Rhinoconjunctivitis Quality of Life Questionnaire (PRQLQ, number)^14^ and nasal peak expiratory flow (NPEF, L/min)^15^ were tested on the same day and were done in the same setting for each patient. They were tested once and randomly on different days over the 5 years. NPEF was measured three times in the seated position using a Mini-Wright peak flow meter (Clement Clarke International, Ltd. London, U.K.) connected to an anesthetic face mask. Subsequently, the highest value was eventually selected. The questionnaire was writ by children and under the assistance of their parents.

### Correlation of air pollution and PRQLQ, NPEF

The mean concentrations of each air pollutant within 7 days before the tests were calculated for the study. A higher PRQLQ indicates that patients suffer more discomfort from nasal symptoms (indicating a worse quality of life). Correlations between PRQLQ scores and mean pollutant concentrations were compared to determine the influence of obesity on the quality of life of AR children when they attained higher air pollutant concentrations. A lower NPEF indicates that the patients suffered a higher degree of nasal obstruction and nasal mucosa inflammation. Correlations between NPEF rates and mean pollutant concentrations were also compared to determine the influence of obesity on nasal obstruction and inflammation when they exposed to higher air pollutant concentrations. Potential confounders were adjusted, including age, sex, second-hand smoke exposure, combined with asthma or not and parental occupation. Parental occupation included white-collar (teacher, public official, and professionals, among others), blue-collar (company employee and others), and others (peasants or fishermen, low-income or no fixed job). Mean ambient temperature and relative humidity within 7 days before the test were also adjusted.

### Association between obesity and AR discomfort when children with AR are exposed to higher air pollutant concentrations

To find the association, mean air pollutant concentrations within 7 days before the tests were divided into low and high levels. For example, the concentration range of CO was divided to 0.35–0.60 ppm (low level) and 0.61–0.86 ppm (high level); PM_10_ was divided to 34.0–67.2 μg/m^3^(low level) and 67.3–100.5 μg/m^3^ (high level); and PM_2.5_ was divided to 20.0–40.7 μg/m^3^(low level) and 40.8–61.5 μg/m^3^ (high level). PRQLQ contain 23 items and each item on a scale of 0 (not troubled) to 6 (extremely troubled)^14^. Scales euqal or higher then 4 (quite troubled) were equal to more severe AR symptoms in our clinical experience. Therefore, children whose PRQLQ scores were equal or higher than 92 (sceles 4 multiply by 23 items) were considered as those with obvious AR discomfort on the study day. When children are exposed to high pollutant level as mentioned above, the rates of subjects with obvious AR discomfort (higher PRQLQ scores) were compared between obese and non-obese groups. We aimed to determine the association between obesity and AR discomfort when children with AR are exposed to higher air pollutant concentrations.

### Statistical analysis

All analyses were performed using Statistical Analysis Software (SAS) (version 9.4; SAS Institute Inc, Carey, NC) and PASW Statistics 18 (IBM, Armonk, NY, USA). Chi-square tests were used for categorical variables, and the *t*-test was used for continuous variables. Spearman’s rank correlations were used for the correlation test between PRQLQ scores, NPEF rates, and pollutant concentrations because the seven pollutant concentrations were not normally distributed. Multivariate regression and multivariate logistic regression analysis was used to adjust for the confounding factors. Statistical significance was set at *p* < 0.05 for a two-tailed test. We interpreted the correlation coefficient values with the method reported by Portney and Watkins^16^, as follows: (1) 0.00–0.25 and − 0.25–0.00, no relationship; (2) 0.25–0.5 and − 0.25 to − 0.5, fair relationship (3) 0.5–0.75 and − 0.5 to − 0.75, moderate-to-good relationship; and (4) < 0.75 and < − 0.75, good-to-excellent relationship. Statistical significance was set at *p* < 0.05 for a two-tailed test, and a correlation coefficient of > 0.25 or < − 0.25. This study was approved by the Institutional Review Board of Chung Shan Medical University Hospital, Taichung, Taiwan (ethics approval number: CS15082, CS1-22,040). All experiments were performed in accordance with relevant guidelines and regulations. Informed consent was obtained from all subjects and/or their legal guardian(s).

## Results

### Demographic data and parameters

**Figure 1:**
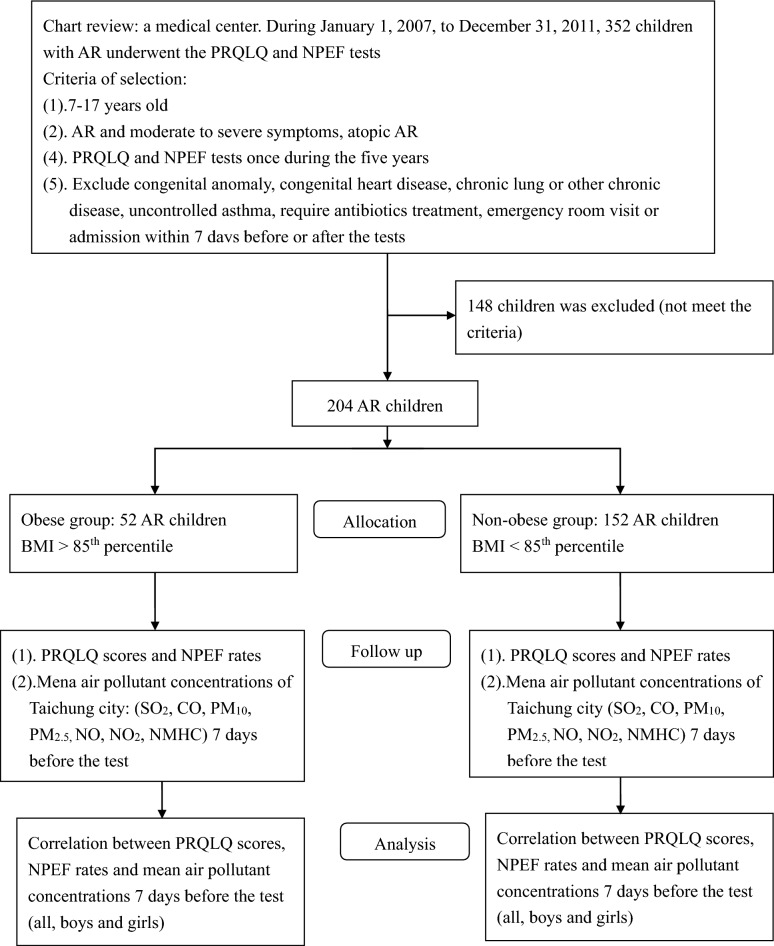
Flow diagram.

The flow diagram is presented in Fig. 1. Table 1 presents the demographic data. During this period, 352 children with AR underwent the PRQLQ and NPEF tests, and 204 children met the criteria of our study. Among them, 52 (25 males and 27 females) and 152 children (80 males and 72 females) were grouped into obese AR and non-obese AR children, respectively. There were no differences between the two groups in age, parental occupation, second-hand smoke exposure, combined with asthma, mean serum total IgE level, and PRQLQ scores. The mean NPEF rates were lower in obese AR children. Data of air pollution, ambient temperature and relative humidity is present in Table 2. There is no difference in these data between the two groups.

**Table 1:** Demographic data, total-IgE, PRQLQ score and PEFR rates.

	Obese AR children (n = 52)	Non-obese AR children (n = 152)	*p*-value
Age in years (mean) ± SD	10.43 ± 2.50	10.52 ± 2.73	0.829
Sex			
Boys	25 (48.1%)	80 (52.6%)	0.571
Girls	27 (51.9%)	72 (47.4%)	
Parental occupation			
White-collar	30 (57.7%)	84 (55.3%)	0.893
Blue-collar	10 (19.2%)	34 (22.4%)	
Others	12 (23.1%)	34 (22.4%)	
Second-hand smoke			
Yes	15 (28.8%)	32 (21.1%)	0.249
Combined with asthma	10 (19.2%)	26 (17.1%)	0.729
Total-IgE (kU/L) ± SD	679.33 ± 698.25	598.35 ± 644.67	0.379*
PRQLQ score (number) ± SD	61.25 ± 32.37	52.69 ± 28.15	0.087*
NPEF level (L/min) ± SD	54.89 ± 34.28	72.46 ± 47.65	0.009*

*p-value: adjust by age, sex, parental occupation, second-hand smoke, combined with asthma.

SD: standard deviation.

**Table 2:** Mean concentration of air pollutants, mean ambient temperature and relative humidity within 7 days before the tests (mean concentration ± standard deviation).

	Obese AR children (n = 52)	Non-obese AR children (n = 152)	*p*-value
SO_2_ (ppb)	3.48 ± 0.58	3.39 ± 0.45	0.217
CO (ppm)	0.63 ± 0.17	0.61 ± 0.14	0.334
PM_10_ (μg/m^3^)	63.34 ± 17.58	59.85 ± 16.46	0.196
PM_2.5_ (μg/m^3^)	40.31 ± 10.40	38.869.34 ±	0.349
NO (ppb)	7.20 ± 2.84	7.07 ± 3.24	0.797
NO_2_ (ppb)	22.89 ± 5.22	21.98 ± 5.37	0.284
NMHC (ppm)	0.32 ± 0.08	0.31 ± 0.07	0.383
Ambient temperature (°C)	22.88 ± 4.28	23.69 ± 4.07	0.220
Relative humidity (%)	71.02 ± 2.16	71.68 ± 2.97	0.141

### Correlation between PRQLQ scores and air pollution

The Spearman’s rank correlation test was used to calculate the Spearman’s rank correlation coefficient (Spearman’s ρ). Table 3 presents the data. In obese AR children, after adjustment by the confounding factors, the PRQLQ scores were statistically positively correlated with mean CO, PM_10_, and PM_2.5_ concentrations within 7 days before the test. The results were: CO Spearman’s ρ = 0.396, *p* = 0.026; PM_10_ Spearman’s ρ = 0.502, *p* = 0.025; PM_2.5_ Spearman’s ρ = 0.506, *p* = 0.022. In contrast, no correlation between PRQLQ scores and the seven pollutants was noted in non-obese AR children. Additionally, regardless of statistical significance, the correlations of the seven pollutants were stronger in obese AR children (with a higher correlation coefficient).

**Table 3:** Correlation between PRQLQ score, NPEF rates and mean air pollutant concentrations (within 7 days before the tests).

	Obese AR children (n = 52)				Non-obese AR children (n = 152)			
	PRQLQ		NPEF		PRQLQ		NPEF	
	Spearman's ρ	*p*-value	Spearman's ρ	*p*-value	Spearman's ρ	*p*-value	Spearman's ρ	*p*-value
SO_2_	0.264	0.345	− 0.255	0.111	0.140	0.644	− 0.179	0.150
CO	0.396	0.026	− 0.379	0.048	0.254	0.300	− 0.208	0.013
PM_10_	0.502	0.025	− 0.429	0.013	0.286	0.063	− 0.162	0.116
PM_2.5_	0.506	0.022	− 0.489	0.005	0.289	0.083	− 0.176	0.041
NO	0.276	0.584	− 0.306	0.071	0.202	0.468	− 0.227	0.050
NO_2_	0.311	0.368	− 0.223	0.346	0.235	0.176	− 0.052	0.923
NMHC	0.319	0.416	− 0.415	0.043#	0.208	0.770	− 0.208	0.049

Spearman's ρ: Spearman’s rank correlation coefficient *p*-value: adjust for sex, age, parental occupation, second-hand smoke exposure, combined with asthma, mean ambient temperature and relative humidity within 7 days before the tests.

When ambient temperature and relative humidity were not added for adjustment (only adjust for sex, age, parental occupation, combined with asthma or not and second-hand smoke exposure), a positive correlation between PRQLQ scores and CO, PM_10_, and PM_2.5_ concentrations were noted in non-obese AR children.

### Correlation between NPEF rates, and air pollution

Table 3 presents the data. In obese AR children, NPEF rates were negatively correlated with CO, PM_10_, PM_2.5_, and NMHC concentrations within 7 days before the test. The results were: CO Spearman’s ρ = − 0.379, p = 0.048; PM_10_ Spearman’s ρ = − 0.429, *p* = 0.013; PM_2.5_ Spearman’s ρ = − 0.489, *p* = 0.005; NMHC Spearman’s ρ = − 0.415, p = 0.043. No correlation was observed between the NPEF rates and the seven air pollutants in non-obese AR children.

### Correlation between PRQLQ scores, NPEF rates, and air pollution in males and females

Table 4 presents the data on obese AR children. In girls with obesity, the PRQLQ scores were statistically positively correlated with SO_2_, PM_10_, and PM_2.5_ concentrations. The results were: SO_2_ Spearman’s ρ = 0.290, *p* = 0.016; PM_10_ Spearman’s ρ = 0.540, *p* = 0.030; PM_2.5_ Spearman’s ρ = 0.517, *p* = 0.024. In boys with obesity, no correlations were found. In girls with obesity, the NPEF rates were statistically negatively correlated with PM_10_ and PM_2.5_ concentrations. The results were: PM_10_ Spearman’s ρ = − 0.532, *p* = 0.013; PM_2.5_ Spearman’s ρ = − 0.561, *p* = 0.016. In boys with obesity, no correlations were found. In non-obese AR children, and in both boys and girls, no correlation was observed between PRQLQ scores, NPEF rates, and air pollutant concentrations.

**Table 4:** Obese AR children, correlation between PRQLQ score, NPEF rates and mean air pollutant concentrations (within 7 days before the tests).

	PRQLQ				NPEF			
	Boys (n = 25)		Girls (n = 27)		Boys (n = 25)		Girls (n = 27)	
	Spearman's ρ	*p*-value	Spearman's ρ	*p*-value	Spearman's ρ	*p*-value	Spearman's ρ	*p*-value
SO_2_	0.325	0.909	0.290	0.016	− 0.227	0.663	− 0.253	0.091
CO	0.359	0.065	0.440	0.111	− 0.182	0.359	− 0.428	0.136
PM_10_	0.473	0.180	0.540	0.030	− 0.381	0.530	− 0.532	0.013
PM_2.5_	0.460	0.149	0.517	0.024	− 0.385	0.284	− 0.561	0.016
NO	0.307	0.547	0.270	0.362	− 0.223	0.206	− 0.310	0.130
NO_2_	0.257	0.247	0.339	0.933	− 0.228	0.382	− 0.216	0.337
NMHC	0.352	0.420	0.307	0.600	− 0.268	0.065	− 0.421	0.323

Spearman's ρ: Spearman’s rank correlation coefficient.

*p*-value: adjust for age, parental occupation, second-hand smoke exposure, combined with asthma, mean ambient temperature and relative humidity within 7 days before the test.

### Association between obesity and AR quality of life when children with AR are exposed to higher air pollutant levels

The results are presented in the Table 5. For example, 122 children with AR were exposed to high CO level (0.60–0.86 ppm) within 7 days before the tests. In obese group, 13 in 33 (39.4%) had obvious AR discomfort (higher PRQLQ scores) when they are exposed to higher CO level. In non-obese group, the rate was 18.0% (16 in 89 subjects). It means that when children with AR were exposed to high CO level, the rats of obvious AR discomfort were higher in obese group, compare to non-obese group (*p*-value = 0.028; Odds Ratio (OR) 3.54, 95% confidence interval (CI) 1.15 ~ 10.92). The rates of obvious AR discomfort were also higher in obese children when they were exposed to higher PM_10_ and PM_2.5_ levels, compare to the rates in non-obese children.

**Table 5:** When children with AR are exposed to higher air pollutant levels, the associations between high PRQLQ score (equal or higher than 92) and obesity.

	Obese AR children	Non-obese AR children	*P*-value	OR (95% CI)
CO (0.60–0.86 ppm)	13/33 (39.4%)*	16/89 (18.0%)*	0.028	3.54 (1.15 ~ 10.92)
PM_10_ (67.2–100.5 μg/m^3^)	12/27 (44.4%)*	14/64 (21.9%)*	0.049	3.26 (1.01 ~ 10.57)
PM_25_ (40.7–61.5 μg/m^3^)	11/28 (39.3%)*	15/76 (19.7%)*	0.044	3.30 (1.03 ~ 10.54)

*p*-value: adjust for sex, age, parental occupation, second-hand smoke exposure, combined with asthma, mean ambient temperature and relative humidity within 7 days before the tests.

*Number of children with high PRQLQ score/number of all children.

OR: Odds Ratio.

## Discussion

A lower NPEF rates indicates a higher degree of nasal obstruction and mucosa inflammation^17,18^. In obese AR children in our study, we find a relationship between higher CO, PM_10_, PM_2.5_, NMHC concentrations and higher degree of nasal obstruction/inflammation (lower NPEF rates). Our study results were compatible with other studies. These studies suggest that air pollution could induce nasal obstruction^3^ and nasal mucosa inflammation^19^. In obese AR children in our study, we also find a relationship between higher CO, PM_10_, PM_2.5_ concentrations and higher AR discomfort or symptoms (higher PRQLQ scores). Therefore, we speculate that, through the induction of nasal obstruction and mucosa inflammation, CO, PM_10_, and PM_2.5_ had a negative impact on nasal discomfort and symptoms in obese AR children. We also found that, in non-obese AR children, there were no correlations between air pollution and AR discomfort/symptoms and nasal obstruction/mucosa inflammation.

When exposed to pollutants, rhinitis symptoms can be exacerbated by immunological (long-term effect) and neurogenic (short-term effect) mechanisms^3^. In our study, air pollution data were gathered within 7 days before the tests. Because of the short duration, the impact of air pollution on AR might have been caused by the neurogenic effects in our study. The trigeminal nerve innervates the nasal cavity. A study indicated that, through the activating of trigeminal nerve endings, air pollution exposure can trigger rhinitis^3,20^. Another study indicated that that obesity can increase the nociceptive activation of the trigeminal system under external stimulation^21^. The abovementioned findings might explain our result, which showed that air pollution had a more detrimental effect on AR when they were obese, compared to those without obesity.

A rats and mouse study found that airborne particulate matter (PM) can be transported via the trigeminal nerve. PM may also induce neurotoxicity, oxidative stress, and neuro-inflammation of nerves^22^. Another study indicated that particles less than 100 nm were observed in human trigeminal ganglia capillaries^23^. This implies that PM could influences the trigeminal nerve and might result in the exacerbating of AR. In children with obesity in our study, we found a stronger correlation between PM_10_, PM_2.5_, PRQLQ (more severe AR symptoms), and NPEF (more severe nasal obstruction and inflammation). The two aforementioned studies by might explain the stronger correlation between PM_10_, PM_2.5_, and AR, compared to those of other air pollutants.

A reduction in the sympathetic tone can cause venous sinusoid dilatation, which can contribute to nasal obstruction symptoms^24^. It was suggested that men have higher sympathetic tone and women have higher parasympathetic autonomic activity^25^. A study indicated that women appear to be more sensitive to intranasal trigeminal stimulation than men^26^. Another study reported a sex-differentiated electrophysiological response to intranasal trigeminal stimuli^27^. These findings could explain the stronger correlation observed between PRQLQ, NPEF, and air pollution (PM_10_, PM_2.5_) in female children with obesity and AR in our study, compared to male children.

Two molecular mechanisms might explain our study results. First, air pollution could induce more nasal inflammation because obese children are in the chronic inflammatory states. Second, air pollution could induce more neuro-inflammation in obese children. Our study results (air pollutants induce higher nasal obstruction/inflammation in obese children) could explain the first speculation. However, the role of neuro-inflammation was not investigated in our study. Future study is necessary.

Ambient temperature and relative humidity was reported to influence the severity of AR^28^. Meteorological conditions including temperature and humidity were also report to be correlated with concentration of air pollutants^29^. Therefore, temperature and humidity could influence both the severity of AR and concentration of air pollutants, and should be adjusted when investigating the association between air pollution and AR. However, most studies investigating the relationship between air pollution and AR in children did not adjust for the two co-factors^30^. The two co-factors were adjusted in our study, so we can find the association between obesity, AR and air pollution more accurate.

Furthermore, our study had some limitations. First, the study was conducted at a single center. Therefore, it cannot reflect the general profile of the entire population owing to selection bias. Second, potential confounders (dietary habits, physical activity, and emotional factors) were not analyzed. Third, only children with moderate to severe AR were assessed in this study. Therefore, future studies including more children with AR from different clinics and areas, patients with mild AR, and gathering more confounding factors such as dietary habits, physical activity, and emotional factors are required.

## Conclusion

The association between air pollution, AR, and obesity remain unclear to date. This study first demonstrated that air pollutant CO, PM_10_, and PM_2.5_ negatively impacted discomfort and symptoms of rhinitis in obese AR children, rather than non-obese AR children. When obese AR children attain higher pollutant concentrations, increased nasal obstruction and inflammation might be the mechanism underlining the present study findings. This suggests that children with obesity and AR should avoid exposure to air pollutants, specifically CO, PM_10_, and PM_2.5_. Although we observed a significant correlation between air pollutants, obesity, and AR in Taiwan, regional differences in air pollution concentration, socioeconomic status, and race could have also altered the outcomes. Therefore, future studies should incorporate more diverse groups of participants with more significant variations in geographical location, socioeconomic status, race, and sex. Biological study to explain the association between AR, obesity and air pollution are required.

## Data Availability

The data are not publicly available due to privacy and ethical. The data presented in this study are available on request from the corresponding author.
